# ﻿Mites of the genus *Bryobia* (Acari, Tetranychidae): taxonomic notes on some species and a diagnostic key to the world species

**DOI:** 10.3897/zookeys.1239.149111

**Published:** 2025-05-20

**Authors:** Jawwad Hassan Mirza, Nasreldeen Ahmed Elgoni, Muhammad Kamran, Fahad Jaber Alatawi

**Affiliations:** 1 Department of Plant Protection, College of Food and Agriculture Sciences, King Saud University, Riyadh 11451, Saudi Arabia King Saud University Riyadh Saudi Arabia

**Keywords:** *Allobia*, *Lyobia*, *neoribis*, *praetiosa*, species groups, subgenera

## Abstract

The present study aimed to develop taxonomic keys to the world species of the genus *Bryobia*, categorized into three subgenera: *Allobia* Livschits & Mitrofanov, *Bryobia* s. str. Koch, and *Lyobia* Livschits & Mitrofanov. Published descriptions, redescriptions, and illustrations of a total of 149 world species were thoroughly analyzed. The taxonomic notes on the status of the species in the species groups of each subgenus are discussed in detail. The variability of morphological characters found among different populations of a species is discussed. As a result, 116 species of the genus *Bryobia* were classified in three diagnostic keys, with 22, 43, and 51 species assigned to the three subgenera *Allobia*, *Bryobia*, and *Lyobia*, respectively. The population of *B.neoribis* Tuttle & Baker from Utah, USA, should be re-identified through type examination due to differences from the original description of the species. Additionally, taxonomic notes are provided on the status of the remaining 33 species, and arguments are provided on suggested synonyms among them.

## ﻿Introduction

The genus *Bryobia* Koch, 1836, is the largest in the subfamily Bryobiinae (Pritchard and Baker 1955) and comprises 149 described species reported globally (Migeon and Dorkeld 2025). These mites are phytophagous and include some of the most notorious pests (Jeppson et al. 1975). The clover mite, *B.praetiosa* Koch, 1836, is a famous member of the genus, infesting different economic fruit, grain, and ornamental crops, and is distributed worldwide (Jeppson et al. 1975).

**Figure 1.**
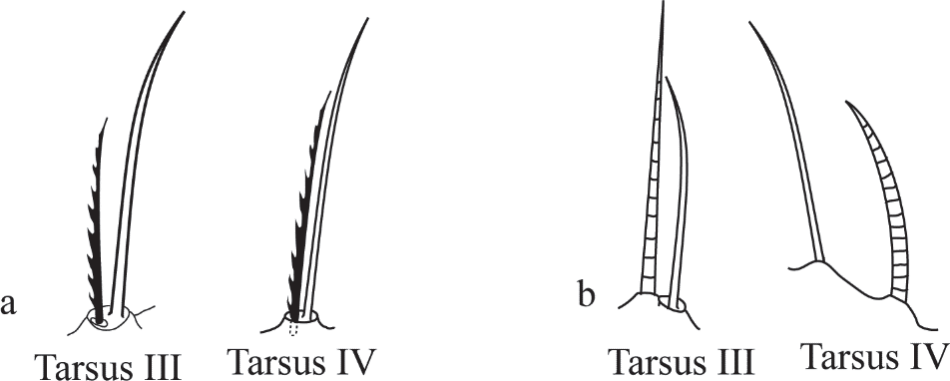
Duplex setae on leg tarsi III and IV **a** duplex setae present on both leg tarsi III-IV in Bryobia (Bryobia) praetiosa Koch, 1836 (redrawn from Livschits and Mitrofanov 1971) **b** duplex setae absent on leg tarsus III in Bryobia (Lyobia) rubrioculus (Scheuten, 1857) (redrawn from Vacante 1983).

Historically, *Bryobia* species were once divided into seven species groups based on the presence of a row of stout setae on leg femur I (Eyndhoven 1956). Livschits and Mitrofanov (1971) introduced a comprehensive analysis of the genus, provided new species synonymies, and, based on the combination of eight morphological characters, proposed five subgenera in the genus, while the sixth subgenus was added by Mitrofanov (1973). Recently, Mirza et al. (2024) comprehensively re-evaluated those morphological characters for generic differentiation and proposed three subgenera in the genus *Bryobia*: *Bryobia* Koch s. str., *Allobia* Livschits & Mitrofanov, and *Lyobia* Livschits & Mitrofanov. These subgenera were diagnosed based on the presence or absence of duplex setae (tactile seta with a sensory solenidion) on leg tarsi III and IV (Fig. 1a, b). Additionally, the species of each subgenus were categorized into three species groups based on the position of the inner sacral setae *f_1_* (Mirza et al. 2024). A total of eight species described by Meyer (1974, 1987), which possess pad-like true claws on leg I, were also discussed over the contradiction with the diagnosis of the tribe Bryobiini (Mirza et al. 2024).

There are various morphological characters that have been misinterpreted (e.g., the position of inner and outer sacrals), while others have been mistakenly considered as differences to distinguish species, rather than as intraspecific variations (i.e., body length, length of propodosomal lobes, number of setae on leg segments, length of leg segments). This raised the issue of species complexes, and the perfect example is the *praetiosa* species complex (Pritchard and Baker 1955). Different regional keys have been published over time from around the world, including those from Africa, Asia, the USSR, the USA, and Europe (Meyer 1974, 1987, 1992; Eyndhoven and Vacante 1985; Livschits and Mitrofanov 1971; Ehara 1999; Auger et al. 2015; Çobanoğlu et al. 2021; Stathakis et al. 2022). However, in the absence of a world key to *Bryobia* species, it would be difficult to grasp the true species identity. This study, based entirely on published literature, aims to distinguish true morphological differences from intraspecific variations to validate species statuses, develop taxonomic keys for the world’s *Bryobia* species within the three subgenera proposed by Mirza et al. (2024), and provide taxonomic notes on the status of certain species.

## ﻿Materials and methods

The published morpho-taxonomic literature of 149 world species of the genus *Bryobia* was collected using the websites of different research journals and spider mite web databases (Migeon and Dorkeld 2006–2025). All the published literature related to the taxonomy and systematics of the *Bryobia* species were equally considered. The classification proposed by Mirza et al. (2024) is followed for the subgenera and species groups. The species descriptions, redescriptions, illustrations, taxonomic revisions, and regionally prepared identification keys were critically investigated to develop three dichotomous keys for the three subgenera of the genus *Bryobia* to identify the species.

## ﻿Results and discussion

**Family Tetranychidae Donnadieu**

**Subfamily Bryobiinae Berlese**

### ﻿Tribe Bryobiini

Taxon classificationAnimaliaTrombidiformesTetranychidae

Reck

06A1320E-5E3F-5193-B1D7-B358FBD12A75

#### Diagnosis.

True claws uncinate and empodium pad-like.

### ﻿Genus Bryobia

Taxon classificationAnimaliaTrombidiformesTetranychidae

Koch, 1836

AD5067DB-4D7D-5585-8173-D3BFC143E719

#### Type species.

*Bryobiapraetiosa* Koch, 1836: 8.

#### Diagnosis

(based on females). As defined by Arabuli et al. (2019) and Mirza et al. (2024).

There are four species, *B.apsheronica* Khalilova, 1953, *B.desertorum* Hassan, Afifi & Nawar, 1986, *B.ribis* Thomas, 1896, and *B.weyerensis* Packard, 1889, not included in any subgenus or species group due to inadequate and insufficient literature, as also reported by Mirza et al. (2024). In the very brief descriptive statements of *B.weyerensis*, the original author provided the two completely different generic names to which this species may belong, “*Bryiobia* ? (or *Penthaleus*)” (Packard 1889). The former three species require re-description based on type examination to be added to the respective subgenus and species group.

### ﻿Subgenus Allobia

Taxon classificationAnimaliaTrombidiformesTetranychidae

Livschits & Mitrofanov, 1971

6AC0446D-2546-5280-BEF5-608E3D400428

#### Type species.

*Bryobiapritchardi* Rimando, 1962: 9.

#### Diagnosis

(based on females). As defined by Mirza et al. (2024).

##### ﻿Key to the 22 species of the subgenus Allobia

Species groups definition is based on Mirza et al. 2024.

**Table:** 

1	Dorsocentral setae *f_1_* present centrally, aligned with other dorsocentral setae, the distance *f_1_*-*f_1_* is always shorter than *f_2_*-*f_2_* (Fig. 2a) *abbatielloi* species group	**3**
–	Dorsocentral setae *f_1_* present laterally or sub laterally	**2**
2	Dorsocentral setae *f_1_* present laterally along the margin and the distance *f_1_*-*f_1_* is always greater than *f_2_*-*f_2_* (Fig. 2b) *pritchardi* species group	**4**
–	Dorsocentral setae *f_1_* present sub laterally, neither aligned with other dorsocentral setae nor present marginally, and the distance *f_1_*-*f_1_* could be shorter or longer than *f_2_*-*f_2_* (Fig. 2c) *deserticola* species group	**21**
3	Propodosoma with distinct, 4 well-developed lobes (Fig. 3a)	***B.* (*A.*) *querci* Hatzinikolis & Panou, 1997**
–	Propodosomal lobes absent (Fig. 3b)	***B.* (*A.*) *abbatielloi* (Smiley & Baker, 1995)**
4	Empodium I with 1 pair of tenent hairs (Fig. 4a)	**5**
–	Empodium I with > 1 pairs of tenent hairs (Fig. 4b)	**16**
5	Genu I with ≤ 6setae	**6**
–	Genu I with 7 or 8 setae	**9**
6	Genu I with 6 setae; femur I with 9 setae	***B.* (*A.*) *beaufortensis* Meyer, 1992**
–	Genu I with 4 or 5 setae	**7**
7	Propodosomal lobes well developed; peritremes ending in an enlarge anastomose (Fig. 5a)	***B.* (*A.*) *marcandrei* Hatzinikolis & Panou, 1996**
–	Propodosomal lobes weakly developed	**8**
8	Peritremes ending in simple bulb (Fig. 5b); genu I with 5 setae	***B.* (*A.*) *ylikiensis* (Hatzinikolis & Emmanouel, 1993)**
–	Peritremes ending in an ovate anastomosis; genu I with 4 setae	***B.* (*A.*) *giannitsensis* Hatzinikolis & Panou, 1996**
9	Median propodosomal lobes well developed; femur III with 3 setae	***B.* (*A.*) *relhaniae* Meyer, 1992**
–	Median propodosomal lobes weakly developed or fused into a single lobe (Fig. 3c, d)	**10**
10	Femur II with ≥ 11 setae	**11**
–	Femur II with 8–10 setae	**13**
11	Propodosoma with 8 setae	**12**
–	Propodosoma with 7 setae; peritremes anastomosed	***B.* (*A.*) *aegyptiacus* (Zaher, Gomaa & El-Enany, 1982)**
12	Peritremes terminate in simple bulb	***B.* (*A.*) *nigromontana* Meyer, 1992**
–	Peritremes terminate in a chamber consisting of a few lobes; stylophore with deep depression	***B.* (*A.*) *geyeri* Meyer, 1974**
13	Femur II with 9 setae	***B.* (*A.*) *caricae* Hatzinikolis & Emmanouel, 1991**
–	Femur II with 8 setae	**14**
14	Tibia III with 7 setae	***B.* (*A.*) *macedonica* Hatzinikolis & Panou, 1996**
–	Tibia III with 9 setae	**15**
15	Tarsus III without solenidion, peritremes elongate anastomose	***B.* (*A.*) *pritchardi* Rimando, 1962**
–	Tarsus III with a solenidion, peritremes simple	***B.* (*A.*) *meyerae* Zaher, Gomaa & El-Enany, 1982**
16	Propodosoma with incomplete reticulation medially; peritremes end in simple bulb	***B.* (*A.*) *angolensis* Meyer, 1987**
–	Propodosoma without reticulation	**17**
17	Peritremes end in small anastomosis	**18**
–	Peritremes end in simple bulb	**20**
18	Median propodosomal lobes fused into a single lobe	**19**
–	Median propodosomal lobes well incised and developed; palp tarsus with 7 setae	***B.* (*A.*) *imbricata* Meyer, 1974**
19	Palp tarsus with 6 setae	***B.* (*A.*) *monechmae* Meyer, 1974**
–	Palp tarsus with 7 setae	***B.* (*A.*) *tuberosa* Meyer, 1974**
20	Femur I with 7 setae; stylophore deeply incised	***B.* (*A.*) *coatesi* Meyer, 1974**
–	Femur I with > 7 setae; stylophore rounded	***B.* (*A.*) *incana* Meyer, 1992**
21	Empodium I with a pair of tenant hairs; peritremes ending in a small anastomosis	***B.* (*A.*) *deserticola* Meyer, 1989**
–	Empodium I with 2 pairs of tenant hairs; peritremes ending in a simple bulb	***B.* (*A.*) *birivularis* Meyer, 1989**

##### ﻿Notes on the species of the subgenus Allobia

The subgenus Allobia includes 28 species (Mirza et al. 2024) although only 22 valid species are included in the key above. Among the remaining six species, five species described by Meyer (1974, 1987) have pad-like true claws on leg I. Mirza et al. (2024) provided a detailed discussion on how this character state contradicts the diagnosis of the Bryobiini tribe. In the present study, the sixth species B. (A.) orycustodia Meyer (in Meyer & Ueckermann, 1989) from the species group *pritchardi* is also considered among those five species of Meyer in which leg I true claws are also pad-like. These six species were not added to the diagnostic key for the time being as this requires an update of the diagnoses of all tribes of subfamily Bryobiinae based on the shape of leg I true claws.

**Figure 2.**
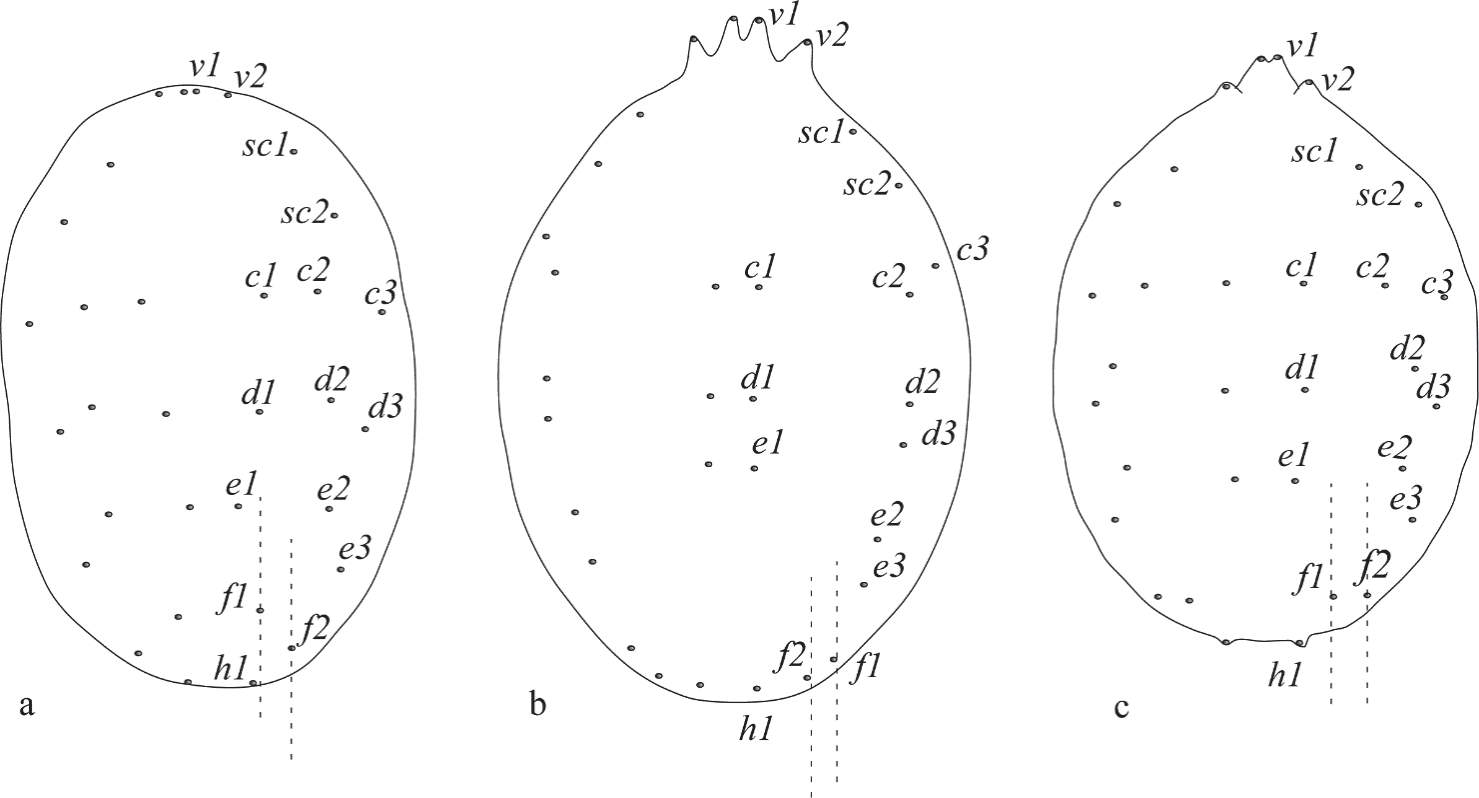
Position of setae *f_1_* and relative distances of *f_1_*–*f_1_* vs *f_2_*–*f_2_* (shown by dashed line) **a** setae *f_1_* present centrally in Bryobia (Allobia) abbatielloi (Smiley & Baker, 1995) (redrawn from Smiley and Baker 1995) **b** setae *f_1_* present laterally in Bryobia (Allobia) pritchardi Rimando, 1962 (redrawn from Rimando 1962) **c** setae *f_1_* present sub laterally in Bryobia (Bryobia) artemisiae Bagdasarian, 1951 (redrawn from Livschits and Mitrofanov 1971).

**Figure 3.**
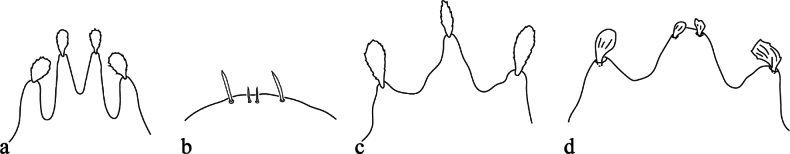
Development of propodosomal lobes **a** well developed in Bryobia (Bryobia) praetiosa Koch, 1836 (redrawn from Livschits and Mitrofanov 1971) **b** absent in Bryobia (Allobia) abbatielloi (Smiley & Baker, 1995) (redrawn from Smiley and Baker 1995) **c** three lobes with median lobes fused in Bryobia (Bryobia) bakeri (Zaher, Gomaa & El-Enany, 1982) (redrawn from Zaher et al. 1982) **d** three lobes in which median lobe is weakly developed in Bryobia (Allobia) geyeri Meyer, 1974 (redrawn from Meyer 1974).

**Figure 4.**
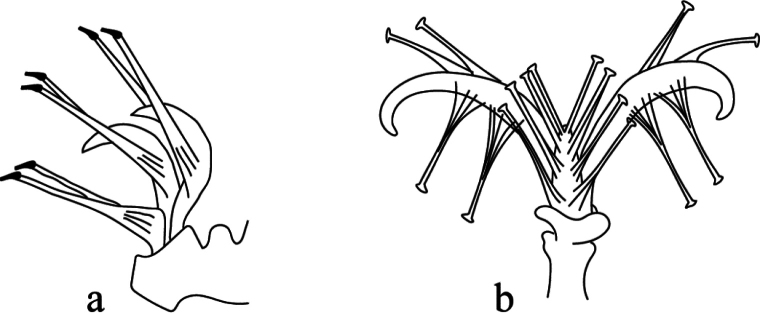
Number of tenent hairs on leg I empodium **a** one pair of tenent hairs in Bryobia (Bryobia) strunkovae Mitrofanov, 1968 (redrawn from Mitrofanov 1968) **b** more than one pair of tenent hairs in Bryobia (Bryobia) borealis Oudemans, 1930 (redrawn from Mathys 1962).

**Figure 5.**
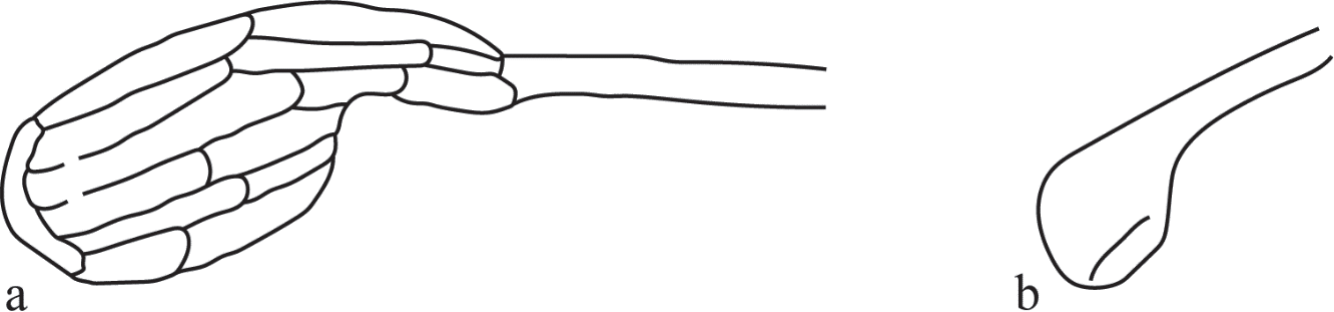
Shape of peritremes **a** enlarged, anastomose in Bryobia (Allobia) marcandrei Hatzinikolis & Panou, 1996 (redrawn from Hatzinikolis and Panou 1996) **b** simple bulb in Bryobia (Allobia) birivularis Meyer, 1989 (redrawn from Meyer and Ueckermann 1989).

##### ﻿Species group *abbatielloi*

There are only two species existing in the species group *abbatielloi*, B. (A.) abbatielloi (Smiley & Baker, 1995) and B. (A.) querci Hatzinikolis & Panou, 1997 (Mirza et al. 2024). The species B. (A.) querci was distinguished by the presence of *f_2_* setae in line with other dorsocentral setae *c_1_*, *d_1_*, and *e_1_* (Hatzinikolis and Panou 1997). This position of seta *f_2_* is incorrectly described in this species, based on the nomenclature of Lindquist (1985). Hence, the seta *f_2_* (outer sacral) described by Hatzinikolis and Panou (1997) is actually seta *f_1_* (inner sacral) and vice versa.

### ﻿Subgenus Bryobia

Taxon classificationAnimaliaTrombidiformesTetranychidae

s. str. Koch, 1836

51B400EB-F78B-5366-8FEA-2C54E6C7D9C5

#### Type species.

*Bryobiapraetiosa* Koch, 1836: 8.

#### Diagnosis

(based on females). As defined by Mirza et al. (2024).

##### ﻿Key to the 43 species of the subgenus Bryobia

Species groups definition is based on Mirza et al. 2024

**Table:** 

1	Fourth pair of dorsocentral setae *f_1_* present centrally, aligned with another 3 pairs of dorsocentral setae *neoephedrae* species group	***B.* (*B.*) *neoephedrae* (Gutierrez & Bolland, 1998)**
–	Fourth pair of dorsocentral setae *f_1_* present sublaterally where the distance *f_1_*-*f_1_* is shorter than *f_2_*-*f_2_ osterloffi* species group	**2**
–	Fourth pair of dorsocentral setae *f_1_* present laterally, along the margin and the distance *f_1_*-*f_1_* always greater than *f_2_*-*f_2_praetiosa* species group	**8**
2	Femur IV with ≥ 5 setae	**4**
–	Femur IV with ≤ 5 setae	**3**
3	Genu II with 5 setae	***B.* (*B.*) *artemisiae* Bagdasarian, 1951**
–	Genu II with 3 setae	***B.* (*B.*) *serifiotica* Hatzinikolis, Papadoulis & Kapaxidi, 2007**
4	Femur IV with 7 setae	***B.* (*B.*) *abyssiniae* Fashing & Ueckermann, 2016**
–	Femur IV with 5 setae	**5**
5	Femur III with 4 or 5 setae	**6**
–	Femur III with 6 or 7 setae	**7**
6	Genu II with 8 setae	***B.* (*B.*) *petrilunara* Meyer, 1987**
–	Genu II with 5 or 6 setae	***B.* (*B.*) *burkei* Meyer, 1987**
7	Genu IV with 6 setae	***B.* (*B.*) *osterloffi* Reck, 1947**
–	Genu IV with 4 or 5 setae	***B.* (*B.*) *variabilis* Manson, 1967**
8	Propodosoma with 7 setae	***B.* (*B.*) *bakeri* (Zaher, Gomaa & El-Enany, 1982)**
–	Propodosoma with 8 setae	**9**
9	Femur IV with 2 or 3 setae	**10**
–	Femur IV with > 3 setae	**13**
10	Genu I with 8 setae	***B.* (*B.*) *meteoritica* Hatzinikolis & Panou, 1996**
–	Genu I with 4 setae	**11**
11	Femur II with 6 setae	***B.* (*B.*) *reckiana* Mitrofanov & Strunkova, 1968**
–	Femur II with 5 setae	**12**
12	Genu III with 3 setae	***B.* (*B.*) *montana* Mitrofanov, 1973**
	**B. (B.) nitrariae He & Tan, 1993**
–	Genu III with 2 setae	***B.* (*B.*) *tadjikistanica* Livschits & Mitrofanov, 1968**
13	Femur IV with 6 setae	**14**
–	Femur IV with < 6 setae	**17**
14	Genu II with 6 setae	**16**
–	Genu II with 4 or 5 setae	**15**
15	Femur I with 18 setae; genu I with 6 setae	***B.* (*B.*) *xiningensis* Ma & Yuan, 1981**
–	Femur I with ≥ 20 setae; genu I with > 6 setae	***B.* (*B.*) *vasiljevi* Reck, 1953**
16	Dorsal integument densely granulates without striae	***B.* (*B.*) *agioriticus* Hatzinikolis & Emmanouel, 1996**
–	Propodosoma with irregular discontinuous fine striae, hysterosoma mostly transverse with irregular fine striae medially	***B.* (*B.*) *alberensis* Auger & Migeon, 2023 (in Auger et al. 2023)**
17	Genu II with 7 or 8 setae	**38**
–	Genu II with < 7 setae	**18**
18	Femur I with ≥ 14 setae	**21**
–	Femur I with ≤ 13 setae	**19**
19	Empodium I with a pair of tenant hairs	**20**
–	Empodium I with > 1 pair of tenant hairs; dorsocentral setae *c_1_* and *d_1_* crossing basis of next setae	***B.* (*B.*) *hengduanensis* Wang & Cui, 1991**
20	Tibiae I and II with 11 or 12 and 9 setae, respectively	***B.* (*B.*) *strunkovae* Mitrofanov, 1968**
–	Tibiae I and II with 16 and 8 setae, respectively	***B.* (*B.*) *ziziphorae* Strunkova & Mitrofanov, 1983**
21	Tibia I with ≥ 21 setae	***B.* (*B.*) *macrotibialis* Mathys, 1962**
–	Tibia I with ≤ 20 setae	**22**
22	Dorsal setae *c_2_* and *c_3_* are in the same horizontal line	**27**
–	Dorsal setae *c_2_* and *c_3_* distinctly not in the same horizontal line	**23**
23	Tarsi III and IV each with 13 setae	***B.* (*B.*) *gigas* Auger, Arabuli & Migeon, 2014**
–	Tarsi III and IV each with > 13 setae	**24**
24	Genua III and IV with 3 and 4 setae, respectively	***B.* (*B.*) *qilianensis* Ma & Yuan, 1981**
–	Genua III and IV each with 6 setae	**25**
25	Femora III and IV each with 4 or 5 setae	**26**
–	Femora III and IV with 7 and 5 setae, respectively	***B.* (*B.*) *latisetae* Wang, 1985**
26	Femora III and IV each with 4 setae	***B.* (*B.*) *exserta* Wang, 1985**
–	Femora III and IV each with 5 setae	***B.* (*B.*) *graminum* (Schrank, 1781)**
	***B.* (*B.*) *monticola* Wang, 1985**
27	Genua I and II with 4 and 3 setae, respectively	**28**
–	Genu I with 7 or 8 setae, genu II with 5 or 6 setae	**29**
28	Stylophore anteriorly rounded, true claws of leg II-IV with 2 rows of tenent hairs ………………	***B.* (*B.*) *magallanica* Gonzalez, 1977**
–	Stylophore anteriorly slightly notched, true claws of leg II-IV with 4–8 tenent hairs	***B.* (*B.*) *glacialis* Berlese, 1913**
29	Tibia I with 12–16 setae	**32**
–	Tibia I with 17–20 setae	**30**
30	Tarsus I with 20 setae	**31**
–	Tarsi I and II with 31 and 19 setae respectively	***B.* (*B.*) *qinghaiensis* Ma & Yuan, 1981**
31	Femur I with 23 setae; tarsus II with 15 setae	***B.* (*B.*) *cyclamenae* Hatzinikolis & Panou, 1996**
–	Femur I with 19 setae; tarsus II with 18 setae	***B.* (*B.*) *platani* Hatzinikolis & Panou, 1997**
32	Empodium I with 2 rows of tenant hairs	***B.* (*B.*) *borealis* Oudemans, 1930**
–	Empodium I with a pair of tenant hairs	**33**
33	Dorsal body setae palmate (Fig. 6a); femur IV with 4 setae; tarsus IV with 14 setae	***B.* (*B.*) *fuegina* Gonzalez, 1977**
–	Dorsal body setae not as above	**34**
34	Propodosoma without lateral projection; tarsal claws II-IV each with > 1 pair of tenant hairs	**35**
–	Propodosoma with lateral projection	**36**
35	Tarsus I with a pair of tenent hairs	***B.* (*B.*) *cagani* Çobanoğlu, Ueckermann & Cilbircioğlu, 2021**
–	Tarsus I with > 1 pair of tenent hairs	***B.* (*B.*) *urticae* Sayed, 1946**
36	Stylophore rounded	***B.* (*B.*) *praetiosa* Koch, 1836**
		***B.* (*B.*) *kissophila* Eyndhoven, 1955**
–	Stylophore notched	**37**
37	Tibia I with 14–16 setae; femur II with ≥ 10 setae	***B.* (*B.*) *watersi* Manson, 1967**
–	Tibia I with 13 setae; femur II with 8 setae	***B.* (*B.*) *attica* Hatzinikolis & Emmanouel, 1990**
38	Genu II with 7 setae	***B.* (*B.*) *emmanoueli* Hatzinikolis & Panou, 1996**
–	Genu II with 8 setae	***B.* (*B.*) *nikitensis* Livschits & Mitrofanov, 1969**

##### ﻿Notes on the species of the subgenus Bryobia

The subgenus Bryobia includes 53 species (Mirza et al. 2024), although only 43 species are included in the key above. Among the remaining ten, two species belong to the species group *praetiosa*, *B.geigeriae* Meyer, 1974, and *B.karooensis* Meyer, 1974, which are excluded from the key due to ambiguity in the leg I true claw morphology as debated by Mirza et al. (2024). The two species B. (B.) calida Karg, 1985 and B. (B.) lagodechiana Reck, 1953 could not be assigned to any species group due to insufficient information available on the position of the inner sacral seta *f_1_*. The status of the remaining six species excluded from the above key is discussed below.

**Figure 6.**
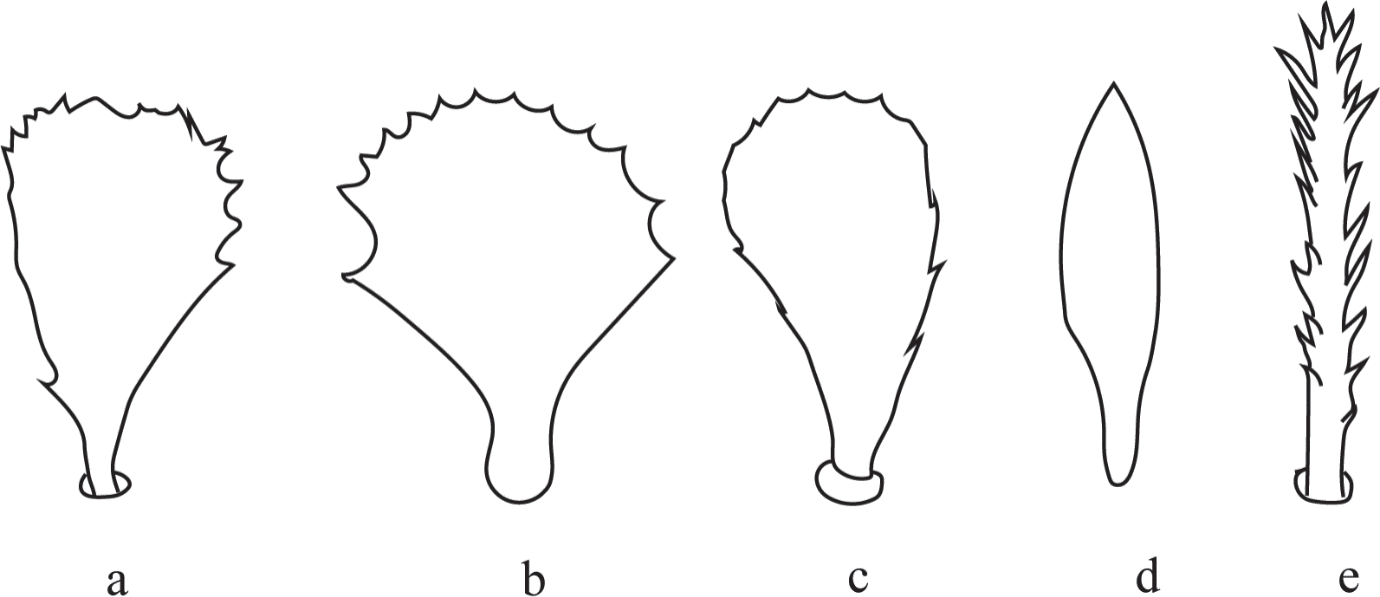
Shape of dorsal setae in adult female **a** palmate in Bryobia (Lyobia) alveolata Auger & Flechtmann, 2009 (redrawn from Auger et al. 2009) **b** fan-shaped in Bryobia (Lyobia) kakuliana Reck, 1956 (redrawn from Livschitz and Mitrofanov 1971) **c** spatulate in Bryobia (Allobia) birivularis Meyer, 1989 (redrawn from Meyer and Ueckermann 1989) **d** lanceolate in Bryobia (Lyobia) gushariensis Livschits & Mitrofanov, 1971 (redrawn from Livschits and Mitrofanov 1971) **e** setiform serrate in Bryobia (Lyobia) cinereae Auger & Migeon, 2014 (redrawn from Auger and Migeon 2014).

##### ﻿Species group *praetiosa*

The species B. (B.) montana Mitrofanov, 1973 was originally described from Tadjikistan on the host plant *Astragalus* sp., while the species B. (B.) nitrariae He & Tan, 1993 was reported from China on the host plant *Nitrariasibirica*. These two species are similar in all morphological characters, including leg chaetotaxy. The only difference is in the number of setae on tarsus I for both species, 20 vs 18, respectively. The descriptions of both species provided leg setal counts as the total number, including sensory and tactile setae. It is important to note that He and Tan (1993) differentiated B. (B.) nitrariae from B. (B.) tadjikistanica Livschits & Mitrofanov, 1968, which is also morphologically close to B. (B.) montana. The two species, B. (B.) tadjikistanica and B. (B.) montana, share the same type locality, Tadjikistan. There are also minor differences between B. (B.) nitrariae and B. (B.) tadjikistanica in the shape of their spermathecae and true claws. The two species B. (B.) montana and B. (B.) nitrariae key out near each other. Examining the type specimens would help to clarify their statuses.

The three species, B. (B.) graminum (Schrank, 1781), B. (B.) monticola Wang, 1985, and B. (B.) neopraetiosa Meyer, 1974 are also morphologically close. They have been reported from Germany (on Poaceae sp.), China (on Poaceae sp.), and South Africa (on multiple hosts), respectively. The leg chaetotaxy for B. (B.) neopraetiosa is neither described nor illustrated in detail (except for femur I, genua I and II, and tibia I), while that of B. (B.) graminum and B. (B.) monticola has few variations on leg tarsal segments. Based on the available descriptions, re-descriptions, and illustrations, it could be suggested that B. (B.) monticola and B. (B.) neopraetiosa should be synonymized with B. (B.) graminum. Similarly, the species B. (B.) exserta Wang, 1985 was reported from China on *Artemisia* sp. and was distinguished from B. (B.) praetiosa Koch, 1836 based on minor morphological variations, including body length, propodosomal lobe lengths, leg genu I segment comparative lengths. Bryobia (B.) exserta also morphologically resembles the three species discussed above. It is impossible to decide the synonymy of B. (B.) exserta, whether it should be synonymized with B. (B.) graminum or B. (B.) praetiosa. The species B. (B.) praetiosa is the type species of the genus *Bryobia*, while B. (B.) graminum, one of the oldest species described, was moved to the genus *Bryobia* by Oudemans (1929). Mitrofanov et al. (1987) synonymized B. (B.) praetiosa with B. (B.) graminum, but previously, Pritchard and Baker (1955) considered synonymizing B. (B.) praetiosa with B. (B.) graminum and suggested further detailed studies. However, these two species still remain valid (Migeon and Dorkeld 2025).

Two species, B. (B.) qinghaiensis Ma & Yuan, 1981 and B. (B.) yunnanensis Ma & Yuan, 1981, are described from China, from the Palearctic and Oriental regions, respectively. They are morphologically similar to each other, apart from some setal variations on leg tarsal and tibial segments, and were differentiated from B. (B.) praetiosa and B. (B.) qinghaiensis, respectively, based on a few minor differences. These species resemble B. (B.) praetiosa, the type of the genus. Note that the concept of apraetiosa species complex still exists, and there are a considerable numbers of populations described under the name of *praetiosa*, or otherwise, from different localities of the world. Each of those descriptions and illustrations provided various degrees of chaetotaxies and body measurements, which further complicate the true identification of B. (B.) praetiosa. Pritchard and Baker (1955) provided an excellent debate on the overall situation of the *praetiosa* complex. It appears that this complex and its synonyms will continue to grow.

The species B. (B.) batrae Baker & Tuttle, 1994 was described from the USA, occurring on the host plant *Stellariamedia*. This species cannot be added to the key as it was very briefly described and illustrated. Baker and Tuttle (1994) also did not compare it with any related species. The species B. (B.) japonica Ehara & Yamada, 1968, also cannot be included as it was also very briefly described. The authors did compare it with B. (B.) sarothamni and B. (B.) tadjikistanica based on the absence of dorsal lobes. These two species belong to the subgenus Allobia (Bryobia) (Mirza et al. 2024).

Tuttle and Baker (1976) described B. (B.) neoribis with a duplex on both leg tarsi III and IV. However, their 1994 original description of the species on *Acernegundo* from Utah, USA stated an absence of duplex on leg tarsus IV. Based on the current designations, the latter species/population belongs to the subgenus Lyobia (Bryobia). The authors further stated that this species was similar to the European B. (B.) ribis Thomas, 1896. The latter is poorly described and has been suggested as a synonym of B. (B.) praetiosa (Pritchard and Baker 1955). This population of B. (B.) neoribis should be reidentified based on type material examination to reach a valid species designation.

The two species, B. (B.) neoribis sensu Tuttle and Baker (1976) and B. (B.) ribis are morphologically close. Mathys (1957) provided detailed morphological analysis and bioecological aspects of the species B. (B.) ribis and other *Bryobia* species found in the French part of Switzerland. The species B. (B.) neoribis was differentiated from B. (B.) ribis based on the number of setae on the femur I (24 vs 16) and variations in body and setal lengths. Tuttle and Baker (1976) did not provide a comprehensive description of the species, preventing detailed comparison and validation with other *Bryobia* species. Similarly, there is no detailed description and illustration of B. (B.) ribis. Mathys (1957) stated that complementary morphological differences could be found in the larval stage of B. (B.) ribis. This raises doubts over the validity of B. (B.) neoribis as only the female stage was briefly described. It would require a comprehensive set of specimens from the type locality to validate the status of the B. (B.) neoribis. For the time being, both species are excluded from the diagnostic key.

The species B. (B.) xizangensis Wang, 1985 was described from China from an unknown host plant. This species was originally differentiated from B. (L.) longisetis Reck, 1947 and was described with one or two pairs of tenent hairs on leg empodium I. Based on the findings of the present study, this species could be morphologically close to B. (B.) hengduanensis Wang & Cui, 1991 due to one pair of tenent hairs present on empodium I, but differentiated based on the length of dorsal body hairs, short vs long, crossing the bases of setae next in line, respectively. Considering the two pairs of tenent hairs on empodium I, B. (B.) xizangensis is similar to B. (B.) ziziphorae Strunkova & Mitrofanov, 1983, but is easily differentiated based on the development of propodosomal lobes, strongly developed and deep incision between the inner and outer lobes vs. weakly developed with small lobes, respectively.

### ﻿Subgenus Lyobia

Taxon classificationAnimaliaTrombidiformesTetranychidae

Livschits & Mitrofanov, 1971

3549F329-9397-5DF8-BFD9-A38FC2C2D12F

#### Type species.

*Bryobiarubrioculus* (Scheuten) 1857: 104.

#### Diagnosis

(based on females). As defined by Mirza et al. (2024).

##### ﻿Key to the 51 species of the genus *Lyobia*

Species groups definition is based on Mirza et al. 2024.

**Table:** 

1	Dorsocentral setae *f_1_* present centrally, aligned with dorsocentral setae *c_1_eurotiae* species group	**3**
–	Dorsocentral setae *f_1_* present laterally or sublaterally	**2**
2	Dorsocentral setae *f_1_* present sublaterally, distance *f_1_*-*f_1_* shorter than *f_2_*-*f_2_sarothamni* species group	**4**
–	Dorsocentral setae *f_1_* present laterally, distance *f_1_*-*f_1_* longer than *f_2_*-*f_2_rubrioculus* species group	**10**
3	Dorsal body setae sit on distinct tubercles; tibia II with 6 tactile setae	***B.* (*L.*) *pamirica* Mitrofanov, 1973**
–	Dorsal body setae sit on indistinct tubercles; tibia II with 9 tactile setae	***B.* (*L.*) *eurotiae* Mitrofanov, 1973**
4	Leg empodium I with 1 pair of tenent hairs	***B.* (*L.*) *chrysocomae* Meyer, 1974**
–	Leg empodium I with > 1 pair of tenent hairs	**5**
5	Dorsocentral setae longer than the distance to bases of setae next in line	**6**
–	Dorsocentral setae distinctly shorter than the distance to bases of setae next in line	**7**
6	Palp of tarsus distinctly longer than tibial claw palp	***B.* (*L.*) *perinsignis* Eyndhoven & Vacante, 1985**
–	Palp of tarsus equal to tibial claw palp	***B.* (*L.*) *nasrvasensis* Bagdasarian, 1960**
7	Stylophore rounded anteriorly	**8**
–	Stylophore emarginate anteriorly	**9**
8	Dorsal body setae lanceolate, broad distally	***B.* (*L.*) *sarothamni* Geijskes, 1939**
–	Dorsal body setae slender	***B.* (*L.*) *annatensis* Manson, 1967**
9	Palptibial claw bidentate	***B.* (*L.*) *polymorpha* Auger & Migeon, 2023 (in Auger et al. 2023)**
–	Palptibial claw simple	***B.* (*L.*) *spica* Pritchard & Keifer, 1958**
10	Propodosoma with reticulation pattern	**11**
–	Propodosoma without reticulation pattern	**14**
11	Dorsal body setae fan-shaped (Fig. 6b) or spatulate or subspatulate (Fig. 6c)	**12**
–	Dorsal body setae palmate; opisthosoma with 7 large oval dimple-like depressions with rounded reticulations	***B.* (*L.*) *alveolata* Auger & Flechtmann, 2009**
12	Opisthosoma with 3 pairs of oval depressions	**13**
–	Opisthosoma without oval depressions; empodium I with 2 rows of tenent hairs	***B.* (*L.*) *dianthi* Mitrofanov & Sharonov, 1983**
13	Propodosoma with 2 large oval lateral depressions; empodium I with 3 pairs of tenent hairs	***B.* (*L.*) *hadizeni* Barbar, Parker & Auger, 2022**
–	Propodosoma without oval depressions; empodium I with 1 pair of tenent hairs	***B.* (*L.*) *mercantourensis* Auger & Migeon, 2014**
14	Most of dorsal body setae spatulate	**15**
–	Dorsal body setae not as above	**38**
15	Empodium I with 1 pair of tenent hairs	**16**
–	Empodium I with ≥ 1 pairs of tenent hairs	**25**
16	Dorsocentral setae *c_1_* and *d_1_* elongate crossing half distance between next setae or reaching basis of next setae	**17**
–	Dorsocentral setae *c_1_* and *d_1_* short not crossing half distance between next setae	**18**
17	First and second pairs of propodosomal setae *v_1_* and *v_2_* equal in length; *c_1_* and *d_1_* reaching or almost reaching bases of next setae	***B.* (*L.*) *dubinini* Bagdasarian, 1960**
–	First pair of propodosomal setae *v_1_* distinctly shorter than second pair *v_2_*; *c_1_* and *d_1_* reaching half distance between next setae	***B.* (*L.*) *longisetis* Reck, 1947**
18	Stylophore notched	**19**
–	Stylophore rounded	**23**
19	Second and fourth pairs of propodosomal setae *v_2_* and *sc_2_* spatulate	**20**
–	Second and fourth pairs of propodosomal setae *v_2_* and *sc_2_* palmate; tibia I with 15 setae	***B.* (*L.*) *kassioticus* Hatzinikolis & Panou, 1997**
20	Area posterior seta *e_1_* with U-shaped striation; propodosoma granulate with irregular longitudinal striae	***B.* (*L.*) *siliquae* Hatzinikolis & Emmanouel, 1991**
–	Area posterior setae *e_1_* with irregular or transverse striae, not U-shaped	**21**
21	Second pair of propodosomal lobes well developed	**22**
–	Second pair of propodosomal lobes absent or poorly developed; femur II with 10 setae	***B.* (*L.*) *syriensis* Barbar & Auger, 2020**
22	Median propodosomal lobes well developed	***B.* (*L.*) *baroni* Auger, Arabuli & Migeon, 2022**
–	Median propodosomal lobes weakly developed or fused	***B.* (*L.*) *populi* Wang & Zang, 1984**
23	Femur II with 12 setae	***B.* (*L.*) *cerasi* Hatzinikolis & Emmanouel, 1991**
–	Femur II with < 12 setae	**24**
24	Dorsum granulate with irregular striae; femur II with 9 setae	***B.* (*L.*) *dikmenensis* Eyndhoven & Vacante, 1985**
–	Dorsum granulate without striae; femur II with 8 setae	***B.* (*L.*) *piliensis* Hatzinikolis & Emmanouel, 1996**
25	Median propodosomal lobes expanded and slightly overlapping; stylophore rounded	***B.* (*L.*) *berlesei* Eyndhoven, 1957**
–	Median propodosomal lobes not overlapping	**26**
26	First and second pairs of propodosomal setae *v_1_* and *v_2_* are equal in length	**35**
–	First pair of propodosomal setae *v_1_* shorter than second pair *v_2_*	**27**
27	Setae *c_3_* in line with setae *c_1_* and *c_2_*	**28**
–	Setae *c_3_* not in line with setae *c_1_* and *c_2_*	**31**
28	Tibia I with 9 setae; genua I and II each with 4 setae	***B.* (*L.*) *nothofagi* Gonzalez, 1977**
–	Tibia I with 12 setae or more; genua I and II each with ≥ 4 setae	**29**
29	Tibia I with 2 sensory setae	**30**
–	Tibia I with 3 sensory setae; femora II and III with 8 and 5 setae, respectively	***B.* (*L.*) *mirmoayedii* Khanjani, Gotoh & Kitashima, 2008**
30	Femora II and III with 10 and 7 setae, respectively	***B.* (*L.*) *cooremani* Eyndhoven & Vacante**,
–	Femora II and III with 7 and 4 setae, respectively	***B.* (*L.*) *vaneyndhoveni* Vacante, 1983**
31	Setae *c_3_* below setae *c_2_*	***B.* (*L.*) *eharai* Pritchard & Keifer, 1958**
–	Setae *c_3_* above setae *c_2_*	**32**
32	Tarsus I with 8 sensory setae	**33**
–	Tarsus I with 7 sensory setae; genu I with 6 setae	**34**
33	Femora I and IV with 13–15 and 4 or 5 setae, respectively	***B.* (*L.*) *cavalloroi* Vacante & Eyndhoven, 1986**
–	Femora I and IV with 10–12 and 6 setae, respectively	***B.* (*L.*) *ulicis* Eyndhoven, 1959**
34	Dorsum integument granulate with irregular striae	***B.* (*L.*) *pyrenaica* Eyndhoven & Vacante, 1985**
–	Dorsum integument granulated	***B.* (*L.*) *strombolii* Vacante, 1983**
35	Setae *c_3_* located above setae *c_2_*	**36**
–	Setae *c_3_* located below setae *c_2_*; empodia I with 2 pairs of tenent hairs	***B.* (*L.*) *chongqingensis* Ma & Yuan, 1981**
36	Genu I with 7 or 8 setae; femora II and III with 9 and 7 setae, respectively	***B.* (*L.*) *aetnensis* Vacante, 1983**
–	Genu I with < 6 setae	**37**
37	Genu I with 6 setae; femur III with 6 setae	***B.* (*L.*) *dekocki* Eyndhoven & Vacante, 1985**
–	Genu I with 4 or 5 setae; femur III with 7 setae	***B.* (*L.*) *vandaelei* Vacante, 1983**
38	Dorsal body setae palmate or fan-shaped	**39**
–	Dorsal body setae lanceolate or slender	**46**
39	Peritremes with simple bulb distally	**40**
–	Peritremes anastomose distally	**42**
40	Dorsal body setae palmate; empodium I with 1 pair of tenent hairs	***B.* (*L.*) *convolvulus* Tuttle & Baker, 1964**
–	Dorsal body setae fan-shaped	**41**
41	Empodium I with 1 pair of tenent hairs	***B.* (*L.*) *oblonga* Livschits & Mitrofanov, 1968**
–	Empodium I with ≥ 1 pairs of tenent hairs	***B.* (*L.*) *kakuliana* Reck, 1956**
42	Dorsocentral setae *c_1_*-*c_1_*, *d_1_*-*d_1_*, *e_1_*-*e_1_* very close to each other	***B.* (*L.*) *angustisetis* Jakobashvili, 1958**
–	Dorsocentral setae *c_1_*-*c_1_*, *d_1_*-*d_1_*, *e_1_*-*e_1_* widely spaced	**43**
43	Femur I with 23 setae; tibia I with 14 setae	***B.* (*L.*) *parietariae* Reck, 1947**
–	Femur I with not more than 21 setae	**44**
44	Tibia I with 12 setae	***B.* (*L.*) *centaureae* Livschits & Mitrofanov, 1972**
–	Tibia I with 15–16 setae	**45**
45	Femur II with 9 setae	***B.* (*L.*) *rubrioculus* (Scheuten, 1857)**
–	Femur II with 11 setae	***B.* (*L.*) *tiliae* (Oudemans, 1928)**
46	Dorsal setae lanceolate (Fig. 6d)	**47**
–	Dorsal setae slender, long at least reaching (Fig. 6e), empodium I with 2 rows of tenent hairs	***B.* (*L.*) *cinereae* Auger & Migeon, 2014**
47	Tibiae III-IV each with 9 setae	**48**
–	Tibiae III-IV each with < 9 setae; femur I with 12 setae or fewer	**49**
48	Femur I with 20 or 22 setae	***B.* (*L.*) *gushariensis* Livschits & Mitrofanov, 1972**
–	Femur I with 13 or 18 setae	***B.* (*L.*) *obihsaphedi* Mitrofanov, 1968**
49	Femur I with 12 setae; tibiae III-IV each 7 setae	***B.* (*L.*) *livschitzi* Mitrofanov & Strunkova, 1968**
–	Femur I with 8 or 9 setae; tibiae III-IV each with < 7 setae	**50**
50	Setae on femora I-IV 9–7–5–3; tibiae III-IV each with 4 setae	***B.* (*L.*) *astragali* Strunkova & Mitrofanov, 1983**
–	Setae on femora I-IV 8–7–4–2; tibiae III-IV with 3 and 5 setae, respectively	***B.* (*L.*) *bucharica* Strunkova & Mitrofanov, 1983**

##### ﻿Notes on the species of the subgenus Lyobia

The subgenus Lyobia includes 58 species (Mirza et al. 2024) but the key to only 51 species is provided above. The species B. (L.) ericoides Meyer, 1974, belonging to the species group *eurotiae*, is excluded from the key due to leg I true claw morphology. The status of the remaining six species, all belonging to the species group *rubrioculus*, are discussed below.

##### ﻿Species group *eurotiae*

The two species in this species group B. (L.) eurotiae Mitrofanov, 1973 and B. (L.) pamirica Mitrofanov, 1973, are morphologically similar and share the type host plant (*Eurotia* sp.), type locality (Tadjikistan), and date of collection (23 July 1967). These two species share most morphological characteristics, including similar body length and width, lacking propodosomal lateral lobes, setae *v_2_* longer than *v_1_*, slender setiform setae, length of leg I equal to body length, number of tenant hairs on leg empodia I-IV, and most of the leg chaetotaxy. The morphological characters which differentiate B. (L.) eurotiae from B. (L.) pamirica include state of propodosomal lobes (completely absent vs inner lobes joined from the middle, forming a cone), dorsal setal tubercles (indistinct vs distinct), leg chaetotaxy of femora I-III (9-7-4 vs 8-6-3), genua I and II (8-5 vs 4-4), and tibia II (9 vs 6), respectively. The differences in leg chaetotaxy mentioned above should be re-examined and could be considered as variations. The original description of B. (L.) eurotiae provides leg chaetotaxy in which tibia I has 24 setae. It appears that the setal count of tibia I was missed, and the setal counts for tarsus I were provided. It could be assumed that there are 24 setae on tarsus I, which was also described for B. (L.) pamirica because the chaetotaxy of tibiae II-IV is similar in both species. Similarly, the setae *f_1_* were described to be present sublaterally in B. (L.) pamirica, while they are illustrated as aligned with dorsocentral setae *c_1_*. Hence, the setae *f_1_* are present centrally or subcentrally in B. (L.) pamirica, similar to B. (L.) eurotiae. Although there is evidence for the possible synonymy of these two species, it is important to re-examine the type specimens to reach a definitive conclusion.

##### ﻿Species group *sarothamni*

There are seven species in this species group (Mirza et al. 2024). The morphology of propodosomal lobes has been described with variations. For instance, B. (L.) sarothamni Geijskes, 1939, was originally described from the Netherlands, with the presence of four propodosomal lobes in the form of tubercles (Geijskes 1939). Pritchard and Baker (1955) distinguished the English population of B. (L.) sarothamni with a complete absence of “cephalic projections”. Baker and Tuttle (1994) reported the presence of the propodosomal projection, where outer ones were as broad as long, and 1/3 as long as the inner pair. This situation is similar to that in the *praetiosa* species complex (in the subgenus Bryobia (B.)). It is recommended to approach the species in this species group with extreme caution, and morphological variations should be completely understood before describing new taxa.

##### ﻿Species group *rubrioculus*

There are 48 species included in this species group (Mirza et al. 2024). The species B. (L.) cinereae Auger & Migeon, 2014 was placed in the species group *sarothamni* (Mirza et al. 2024). However, in the present study, it is included in the species group *rubrioculus* due to the marginal position of sacral *f_1_* and *f_2_* setae. This species is morphologically close to B. (L.) belliloci Auger, Arabuli & Migeon, 2015; however, the morphological differences are debatable. It has been stated that setae *d_1_* clearly surpass the bases of *e_1_* in B. (L.) belliloci (illustrated as just passing) while setae *d_1_* just reach the base of setae *e_1_* in B. (L.) cinereae (Auger and Migeon 2014). There are other morphological characters which were used to differentiate B. (L.) belliloci from B. (L.) cinereae including the depth of the inner lobe incisions (but illustrated as exactly same for both species), peritremal distal enlargement length (both anastomosing but length has 7 μm difference), length of internal seta *l’_1_* on femur I, lengths and shapes of coxal setae *1b* and *1c* (discrepancies in the description and illustrations of B. (L.) belliloci). These characters may reflect variations in the morphologies, especially when both species have the same host plant, *Genistacinerae*, and are both reported from France (Auger and Migeon 2014; Auger et al. 2015). The species B. (L.) belliloci is excluded from the key, and perhaps further studies may suggest it as a junior synonym of B. (L.) cinereae.

The four species B. (L.) tiliae (Oudemans, 1928; Germany), B. (L.) rubrioculus (Scheuten, 1857; Germany), B. (L.) lonicerae Reck, 1956 (Georgia), and *B* (*L.*) *ulmophila* Reck, 1947 (Georgia), are very similar to each other in all morphological aspects including leg morphology. The species B. (L.) rubrioculus has been described and illustrated from different regions of the world and number of species have been synonymized under it (Migeon and Dorkeld 2025). Frommer and Jorgensen (1972) studied the morphological and behavioral variations with host specificity of B. (L.) rubrioculus and distinctly separated this species from B. (L.) praetiosa. The two species B. (L.) lonicerae and B. (L.) ulmophila were morphologically compared with B. (L.) redikorzevi that is considered a synonym of B. (L.) rubrioculus by Frommer and Jorgenson (1972). Wainstein (1960) considered B. (L.) ulmophila as synonym of B. (L.) redikorzevi. The species B. (L.) tiliae was originally described as a type species of the genus *Schmiedleinia* Oudemans, 1928, based on the larval specimens collected from the host plant *Tiliae* sp. in Germany (Oudemans 1928). The genus was later synonymized with the genus *Bryobia*, and the species *tiliae* was considered as the larvae of *B.praetiosa* (Oudemans 1930). Bagdasarian (1957) described the species B. (L.) tiliae from Armenia on the same host plant, *Tiliae* sp. It was later considered a synonym of the species described by Oudemans (1928) (Wainstein 1960). In that synonymy, B. (L.) tiliae was considered to be morphologically close to B. (L.) ulmophila and B. (L.) redikorzevi (Bagdasarian 1957) but distinguished based on the number of setae on leg femur I. Both of the latter two species have been considered as a synonym of B. (L.) rubrioculus. The leg chaetotaxy alone would not be sufficient to confidently validate the identity of the species. In light of this debate, it would be difficult to reach any definitive conclusion regarding the validity of these three species, and their synonymy with B. (L.) rubrioculus requires further investigation.

Eyndhoven and Vacante (1985) described 13 species belonging to the *berlesei* species group, eight of which were described for the first time. Among them, five species B. (L.) pandayi Eyndhoven & Vacante, 1985, B. (L.) pyrenaica Eyndhoven & Vacante, 1985, B. (L.) pelerentsi Eyndhoven & Vacante, 1985, B. (L.) dikmenensis Eyndhoven & Vacante, 1985, and B. (L.) provincialis Eyndhoven & Vacante, 1985, have variable morphological characters. The three species B. (L.) pandayi, B. (L.) pyrenaica, and B. (L.) pelerentsi were considered close to each to other and the differential character designated as “Each species has its own host plant” (Eyndhoven and Vacante 1985: 400). In all other morphological aspects, these three species resemble each other, and it is difficult to differentiate them. The remarks for these species were stated as “For general remarks see *Bryobiapandayi*”. It is important to mention that a species having its own host plant does not necessitate its validity. The species B. (L.) pandayi was reported from *Calicotomespinosa*. The same host plant (*Calicotome* sp.) harbors almost seven *Bryobia* taxa (Migeon and Dorkeld 2025). Interestingly, B. (L.) pelerentsi is also reported from *Calicotome* sp. (Eyndhoven and Vacante 1985). Hence, with this argument, the synonymy of B. (L.) pyrenaica and B. (L.) pelerentsi with B. (L.) pandayi appears undeniable. Similarly, both species, B. (L.) dikmenensis and B. (L.) provincialis are reported from the same host plant (*Genistus* sp.) and were morphologically designated close to each other by Eyndhoven and Vacante (1985). The only morphological difference described was that the second and third dorsocentrals were smaller than the other dorsal body setae in B. (L.) dikmenensis, while of similar length in B. (L.) provincialis. However, this contradicts what has been described for these setae based on 14 specimens (Eyndhoven and Vacante 1985). This places the status of these species as doubtful, and there is an urgent need for re-analysis of the morphological characters of these species.

## ﻿Conclusions

In conclusion, the present study provides a comprehensive taxonomic status of the species of *Bryobia* through detailed literature-based morphological analysis. The diagnostic keys to the majority of *Bryobia* species will undoubtedly prove useful for acarologists. The taxonomic notes on some species and the variability in morphological characters found among different populations of a species deepen our understanding of morphological diversity in the genus. It is important to note that there are four species: *B.apsheronica* Khalilova, 1953, *B.desertorum* Hassan, Afifi & Nawar, 1986, *B.ribis* Thomas, 1896, and *B.weyerensis* Packard, 1889 that are not included in any subgenus or species group due to inadequate and insufficient literature, as also reported by Mirza et al. (2024). The species *B.weyerensis* may not even belong to the family Tetranychidae, while the former three species should be redescribed. Although some species have been suggested to be synonymized with closely related species, a valid taxonomic and systematic decision should be backed and supported by careful examination of the type specimens. In the scenario where types have been lost, a collection should be initiated to revisit the type locality.

## Supplementary Material

XML Treatment for Bryobiini

XML Treatment for Bryobia

XML Treatment for Allobia

XML Treatment for Bryobia

XML Treatment for Lyobia

## ﻿Additional information

### Author ORCIDs

Jawwad Hassan Mirza https://orcid.org/0000-0002-1726-4331

Nasreldeen Ahmed Elgoni https://orcid.org/0009-0007-7698-1377

Muhammad Kamran https://orcid.org/0000-0001-6084-203X

Fahad Jaber Alatawi https://orcid.org/0000-0002-6824-2650

### Data availability

All of the data that support the findings of this study are available in the main text.
